# Prophylactic intravitreal 5-fluorouracil and heparin to prevent proliferative vitreoretinopathy in high-risk patients with retinal detachment: study protocol for a randomized controlled trial

**DOI:** 10.1186/s13063-018-2761-x

**Published:** 2018-07-16

**Authors:** Friederike Schaub, Robert Hoerster, Petra Schiller, Moritz Felsch, Daria Kraus, Marouan Zarrouk, Bernd Kirchhof, Sascha Fauser

**Affiliations:** 1 Department of Ophthalmology, University of Cologne, Kerpener Strasse 62, 50924 Cologne, Germany; 2 MVZ Augenärztliches Diagnostik- und Therapiecentrum Mönchengladbach/Erkelenz GmbH, Ostpromenade 41, 41812 Erkelenz, Germany; 3 Institute of Medical Statistics and Computational Biology (IMSB), University of Cologne, Bachemer Str. 86, 50931 Cologne, Germany; 4 Clinical Trials Center Cologne (CTCC), Gleueler Str. 269D, 50935 Cologne, Germany; 5 F. Hoffmann-La Roche AG, Grenzacherstrasse 124, 4070 Basel, Switzerland

**Keywords:** Proliferative vitreoretinopathy, Retinal detachment, Complication, 5-Fluorouracil, Heparin, Placebo

## Abstract

**Background:**

Proliferative vitreoretinopathy (PVR) is the major cause for postoperative failure after vitreo-retinal surgery for primary rhegmatogenous retinal detachment (RRD). Adjunct pharmaceutical therapy was found to be ineffective once PVR is established. Preliminary data suggest that prevention of PVR yields better functional outcome. So far, there is no standard therapy to prevent PVR.

**Methods/design:**

This is a randomized, double-blind, controlled, multicenter, interventional trial with one interim analysis. High-risk patients for PVR with primary RRD will be allocated equally to the following treatment arms: (a) verum: intraoperative adjuvant application of 5-fluorouracil (5-FU) and low-molecular-weight heparin (LMWH) via intraocular infusion during routine pars plana vitrectomy (PPV) and (b) placebo: routinely used intraocular infusion with balanced salt solution during routine PPV. PVR risk is assessed by non-invasive aqueous flare measurement by using laser flare photometry.

The primary endpoint of the trial is the occurrence of PVR grade CP (C: full-thickness retinal folds or subretinal strands in clock hours; P: located posterior to equator) 1 or higher within 12 weeks after treatment. Secondary endpoints include PVR grade CA (A: located anterior to equator), best corrected visual acuity, number and extent of surgical procedures to achieve retinal re-attachment, and occurrence of drug-related adverse events within 12 weeks.

It is assumed, on the basis of previously published results, that the incidence of PVR grade CP 1 is 35% in the control group and that a reduction by one third would be clinically relevant. Given the sequential design and adjustment for a dropout rate of 5%, a total sample size of 560 patients (280 per group) was calculated to ensure a power of 80% for the confirmatory analysis.

**Discussion:**

The present trial uses intraoperative intravitreal 5-FU and LMWH as a prophylactic therapy in high-risk patients with primary RRD, aiming to reduce the incidence of PVR in the group that receives the trial drug. Using laser flare photometry to identify high-risk patients for PVR, this trial will test the effectiveness of a simple treatment to prevent PVR.

**Trial registration:**

EudraCT no.: 2015-004731-12, registered October 21, 2015; ClinicalTrials.gov Identifier: NCT02834559, registered July 12, 2016. Protocol version: Version 02. Date: September 18, 2016.

**Electronic supplementary material:**

The online version of this article (10.1186/s13063-018-2761-x) contains supplementary material, which is available to authorized users.

## Background

Proliferative vitreoretinopathy (PVR) is the major cause for postoperative failure after surgery for primary rhegmatogenous retinal detachment (RRD). Fibrovascular scars lead to secondary tractional retinal detachments, which require multiple extensive surgical interventions to achieve retinal re-attachment [1, 2]. PVR often leads to blindness. Despite advances in the surgical management of PVR, the visual prognosis is poor and only 11% to 25% of patients achieve a visual acuity of at least 20/200 [3]. So far, there is no standard therapy to prevent PVR. The pathogenesis of PVR involves intravitreal invasion of retinal pigment epithelial cells and retinal glial cells. These cells settle on intraocular interfaces, where they differentiate to contractile myofibroblasts, proliferate, and form fibrovascular scars [1, 4–6].

Several attempts in established PVR using chemotherapeutic agents like 5-fluorouracil (5-FU) and low-molecular-weight heparin (LMWH) (Table 1) or daunomycin have been undertaken to prevent this process; however, none of these treatments gained the status of routine practice [7–10]. The most important reason is lack of improvement of the functional prognosis. Better results are to be awaited if patients at risk of PVR are identified, allowing a preventive treatment regimen.

**Table 1:** Summary of results of previous clinical trials using 5-FU combined with LMWH during pars plana vitrectomy

Reference	Intervention (Dose and duration of trial drug)	Number of treated eyes (*n*) (treatment arm) and follow-up period	Main inclusion criterion (PVR grade)	Endpoint	Results
Asaria et al. [7]	200 μg/mL 5-FU and 5 IU/mL LMWH (in 500 mL intraocular BSS)	*n* = 174 (87) Follow-up: 6 months	High-risk eyes for PVR	Incidence of PVR, re-detachment rate after 6 months, rate of re-operations to achieve stable re-attachment, visual acuity.	Statistically significant reduction in incidence of PVR (12.6% versus 26.4%; *P* = 0.02).
Charteris et al. [11]	200 μg/mL 5-FU and 5 IU/mL LMWH (in 500 mL intraocular BSS - duration 1 h during pars plana vitrectomy with silicone oil tamponade)	*n* = 157 (78) Follow-up: 6 months	PVR grade C	Primary: re-attachment rate following silicone oil removal without necessity of additional surgical interventions. Secondary: visual acuity, formation of epiretinal membranes, glaucoma, cataract, localized retinal detachments.	No statistically significant difference between both groups regarding primary or secondary endpoints.
Wickham et al. [9]	200 μg/mL 5-FU and 5 IU/mL LMWH (in 500 mL intraocular BSS - duration 1 h)	*n* = 641 (342) Follow-up: 6 months	No pre-existing PVR	Primary: re-attachment rate without secondary intervention after 6 months. Secondary: incidence of PVR, visual acuity, postoperative complication rate.	No statistically significant difference between both groups regarding primary endpoint (82.3% versus 86.8%); No statistically significant difference regarding visual acuity overall (*P* = 0.072) but worse outcome in eyes with primarily attached macula (*P* = 0.0091).

Abbreviations: *5-FU* 5-fluorouracil, *BSS* balanced salt solution, *LMWH* low-molecular-weight heparin, *PVR* proliferative vitreoretinopathy

Asaria et al. assessed the adjuvant use of 5-FU (200 μg/mL) combined with LMWH (5 IU/mL) in comparison with placebo in the standard intraocular infusion applied during pars plana vitrectomy (PPV) for RRD repair [7]. In this report, complications or adverse events (AEs) have been limited. The occurrence of 10 postoperative hyphemas was divided equally between the treatment groups. Intraoperative complications included one retinal incarceration and one choroidal hemorrhage. Thus, both of these complications were most likely not due to the medication. In the follow-up period, one patient died of unrelated causes. Altogether, there were no drug-related complications or AEs [7].

Charteris et al. have reported a trial setting similar to that of Asaria et al., using a combination of 5-FU and LMWH but treating eyes with already-established PVR [11]. No significant differences in the number of potential complications due to the adjunct treatment (or the surgery or both) were found between treatment and control groups during the 12-month follow-up period [11]. Complications were reported for 98 patients of both groups, including glaucoma (0 versus 3 controls), hypotony (9 versus 7 controls), keratopathy (5 versus 2 controls), and cataract (21 versus 29 controls), indicating no drug-related AEs [11].

Wickham et al. again investigated the adjuvant effect of 5-FU (200 μg/mL) combined with LMWH (5 IU/mL) in comparison with placebo for RRD repair. No statistically significant difference between both groups with respect to retinal re-attachment rate (82.3% versus 86.8%) and visual acuity outcome (*P* = 0.072) could be detected. However, they reported that adjuvant treatment with 5-FU and LMWH of eyes with primary macula-on RRD led to a significant reduction in the final postoperative visual acuity in comparison with control patients (*P* = 0.0091) [9].

Until now, a major problem has been to identify patients at risk for PVR in order to limit potentially harmful chemotherapy to high-risk patients only and to improve statistical power. So far, high-risk patients for PVR have been determined in a complicated manner based on anamnestic risk factors such as diabetic retinopathy, accompanying uveitis, aphakia, or penetrating ocular trauma [7, 12, 13]. These existing methods for estimation are time-consuming and depend on observers’ judgment, and the area under the receiver operating characteristic (ROC) curve is low [7, 12, 13].

In two independent trials, we have recently identified high-risk patients for PVR by determining protein levels in the anterior chamber using non-invasive laser flare photometry [14, 15]. In eyes with flare values of at least 15 pc/ms (photon counts per millisecond), the odds for developing PVR re-detachment increased 16-fold. Sensitivity and specificity of flare measurements with a cutoff value of 15 pc/ms were 83.3% and 76% (area under the ROC curve of 0.85), respectively. Therefore, non-invasive laser flare photometry has been established as a possible tool for fast and precise estimation of high-risk patients for PVR re-detachments [15]. Conart et al. recently confirmed that preoperative aqueous flare seems to be a major predictive factor for PVR re-detachment [16].

The COCHRANE collaboration has recently reviewed two independent randomized controlled trials using 5-FU and LMWH to prevent PVR (Table 1). The first trial used 5-FU and LMWH in conventionally determined high-risk patients [7], the second trial in unselected patients [9]. High-risk patients benefited from 5-FU and LMWH whereas unselected patients did not [7, 9, 17]. Because results had been inconsistent, the COCHRANE collaboration recommended the use of 5-FU and LMWH in a randomized controlled trial in high-risk patients [17].

### Rationale

Intraoperative intravitreal 5-FU and LMWH as a prophylactic therapy in high-risk patients with primary RRD is used. The rationale of the present trial is to decrease the incidence of PVR in the group that receives the trial drug. With the help of laser flare photometry to determine high-risk patients for PVR, this trial tests the effectiveness of a simple treatment to prevent PVR.

### Investigational medicinal product

LMWH reduces postoperative fibrin and binds fibronectin and growth factors [18], whereas 5-FU inhibits the DNA synthesis and thus proliferation of fibroblasts [19]. As a result of the combined intraocular use during vitrectomy, different stages of the PVR formation process are inhibited and may produce a synergistic effect [17].

The investigational medicinal product (IMP) components, either two verum components (5-FU and LWMH) or two placebo components (balanced salt solution, or BSS), will be injected in the 500 mL intraocular infusion (BSS) in order to constitute the IMP for intravitreal application during PPV. Both verum IMP components will be delivered in the following stock concentrations: component A: dalteparin sodium (water for injection); vial volume: 1.2 mL with a stock concentration of 2500 IU/mL dalteparin in water for injection, volume applied: 1 mL; component B: 5-FU; vial volume: 2.5 mL with a stock concentration of 50 mg/mL 5-FU in water for injection, volume applied: 2 mL. The concentrations of 5-FU and LMWH in 500 mL BSS will be 200 μg/mL and 5 IU/mL, respectively. The resulting ocular irrigating solution will be used in accordance with standard format for each surgical procedure.

### Objectives

The primary objective of the trial is to investigate whether the incidence of PVR can be reduced in high-risk eyes (elevated protein levels in the anterior chamber fluid, laser flare value of at least 15 pc/ms) with RRD by intraoperative adjuvant therapy with 5-FU and LMWH. The secondary objective is to investigate whether adjuvant intravitreal therapy with 5-FU and LMWH affects postoperative outcome parameters and postoperative course in high-risk patients with RRD.

#### Primary endpoint

PVR grade CP 1 or higher (yes/no) within 12 weeks in accordance with the updated classification of proliferative vitreoretinopathy of the Retina Society (1991) [20].

#### Secondary endpoints

- PVR grade CP 1 or higher (yes/no) within 6 weeks
- PVR grade CA 1 or higher (yes/no) within 6 and 12 weeks
- Degree of PVR—PVR grade CA 1–12, PVR grade CP 1–12 (in clock hours)—within 6 and 12 weeks
- Best corrected visual acuity (BCVA) measured by Early Treatment Diabetic Retinopathy Study (ETDRS) charts within 6 and 12 weeks
- Retinal re-attachment after primary intervention (yes/no) within 6 and 12 weeks
- Number of retinal re-detachments and, if present, due to PVR (yes/no) within 6 and 12 weeks
- Number and extent of surgical procedures necessary to achieve retinal re-attachment within 12 weeks
- Occurrence of at least one drug-related AE that affects the study eye (yes/no) within 12 weeks.

### Trial design

This phase III, double-blind, randomized, placebo-controlled study aims to determine whether there is a beneficial effect in using 5-FU and heparin as an adjuvant treatment at the time of initial retinal detachment surgery in patients with a high risk of PVR complicating RRD.

**Fig. 1:**
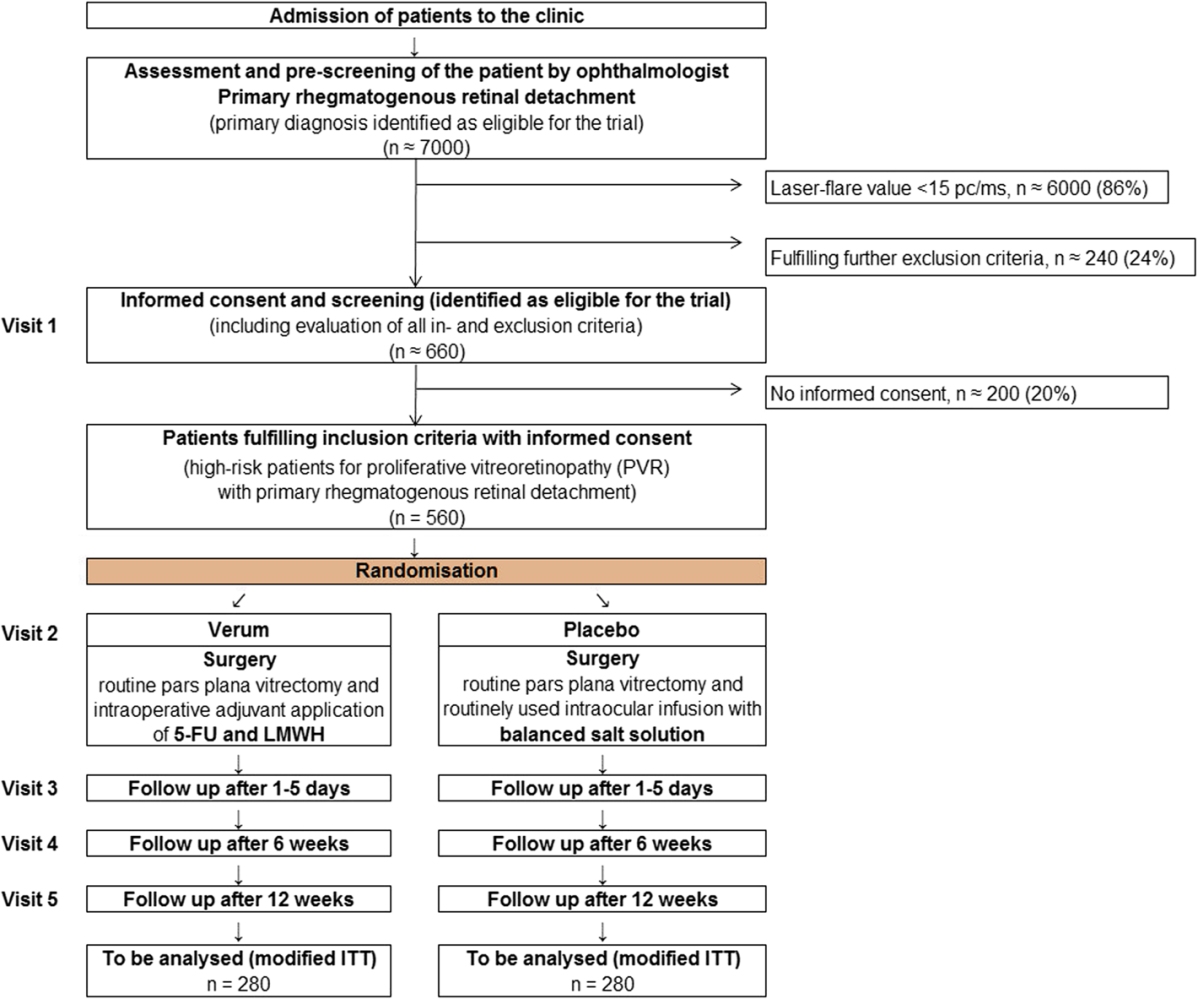
Trial flow. Abbreviations: *5-FU* 5-fluorouracil, *ITT* intention to treat, *LMWH* low-molecular-weight heparin, *pc/ms* photon counts per millisecond

Five hundred sixty patients are planned to be included in the trial. They are to be divided into two equal groups (verum and placebo arm). They are recruited for the study if they satisfy the inclusion and exclusion criteria (Fig. 1). Participants and study team members, including surgeons, are masked to the treatment arm.

## Methods/design

### Study coordination

The study is coordinated by the lead principal investigator (PI) and a delegate. Furthermore, organizational tasks are delegated to a project manager of the Clinical Trials Center Cologne (CTCC).

### Trial sites and study teams

Only specialized vitreo-retinal study centers are eligible for participation in the PRIVENT (Prophylactic Intravitreal 5-Fluorouracil and Heparin to Prevent PVR in High-risk Patients With Retinal Detachment) trial. Pre-study selection visits have been performed by the medical study coordinator and a monitor to select centers on the basis of the number of recruitable patients and the quality of trial support infrastructure. Also, experience in clinical trials, which was a further important prerequisite for participation, has been evaluated during the selection visit.

All study team members of all trial sites have been trained during the initiation visits by the medical coordinator and delegate of the sponsor’s representative and by the monitor. Furthermore, only surgeons with sufficient experience in vitreo-retinal surgery are allowed to perform the PPV in the PRIVENT trial. Their expertise must be proven and is checked by the medical and organizational coordinators. Regular meetings for study staff members and surgeons are organized to discuss potential problems during the trial.

### Participants, interventions, and outcome

#### Study setting

Hospitalized patients and ambulatory care of 13 German trial sites (Appendix). Additional trial sites may still be selected during the study.

#### Eligibility criteria

##### Inclusion criteria

1. Primary rhegmatogenous retinal detachment (<4 weeks) in study eye
2. Scheduled for PPV for retinal detachment repair without combined cataract surgery in study eye
3. Elevated protein levels in anterior chamber fluid (laser flare value of at least 15.0 pc/ms) in study eye
4. Female or male patient of at least 18 years of age
5. Written informed consent.

##### Exclusion criteria

Any of the following will exclude a patient from the trial:

1. Retinal detachment lasting more than 4 weeks in study eye
2. Traumatic retinal detachment in study eye
3. Giant retinal tears in study eye (size of more than 3 clock hours)
4. Visual pre-existing PVR grade C in study eye
5. Retinal dystrophies in study eye
6. Scheduled for combined PPV and cataract surgery for retinal detachment repair in study eye
7. Chronic inflammatory conditions in study eye
8. Active retinal vascular disease in study eye
9. Proliferative diabetic retinopathy in study eye
10. Manifest uveitis in study eye
11. Endophthalmitis in study eye
12. Perforating and non-perforating trauma in study eye
13. Malignant intraocular tumor in study eye
14. Aphakia in study eye
15. Uncontrolled glaucoma or ocular hypertension in study eye (intraocular pressure (IOP) of at least 30 mm Hg despite IOP-lowering therapy)
16. Previous intraocular surgery except uncomplicated cataract surgery with posterior chamber lens implantation in study eye
17. Cataract surgery in study eye not more than 3 months ago
18. Previous retinal procedures—laserpexy, cryopexy, intravitreal gas injection, anti- vascular endothelial growth factor (anti-VEGF), or corticosteroid injection—in study eye not more than 6 months ago
19. Other uncontrolled ophthalmologic disorders
20. Single-eyed patients (BCVA of fellow eye of more than 1.0 log magnification requirement (MAR), less than 0.1 decimal, less than 1/10 tenth, or less than 6/60 Snellen fraction [m])
21. Evidence or history of alcohol, medication, or drug dependency within the last 12 months
22. Evidence or history (within the last 12 months) of neurotic personality, psychiatric illness that requires or required treatment, epilepsy, or suicide risk
23. Systemic disorders not compatible with adjuvant application of 5-FU and LMWH via intraocular infusion or not compatible with the local or general anesthesia
24. Any therapy with immunosuppressant or chemotherapy of not more than 3 months and during the trial period
25. Participation in another trial of IMPs or devices parallel to, or less than 3 months before screening, or previous participation in this trial
26. Known to or suspected of not being able to comply with the protocol
27. Inability to understand the rationale of this trial or the study aim
28. Any dependency of the patient to the investigator or the trial site (e.g. employees with direct involvement in the proposed trial or in other trials under the direction of this investigator or trial site as well as family members of the employees or the investigator)
29. Positive urine pregnancy test, pregnancy, or breastfeeding mother
30. Women of childbearing potential without satisfactory contraception (i.e. hormonal contraceptives for at least 14 days before trial enrolment or intrauterine device; women of childbearing age must be counseled about the use of adequate contraception).

### Interventions

Both groups will receive standard surgical treatment and routine preoperative and postoperative treatment and care comprising standard PPV with or without placement of a scleral buckle. During PPV, the IMP will be administered via intraocular infusion. The verum/placebo containing infusion will be used for a maximum of 60 min. If surgery takes longer, infusion will be changed to normal BSS. Furthermore, retinal breaks will be identified and the following methods will be performed at the operating surgeon’s discretion: retinopexy to retinal breaks and retinectomy edge by cryotherapy or laser, internal limiting membrane peeling, intraocular endodrainage using air, perfluorocarbon, and intraocular tamponade using expanding gas or silicone oil. Applications of intravitreal steroids or cataract extraction with or without intraocular chamber lens implantation are prohibited during primary PPV.

#### Study visits and assessment schedule

Preoperatively, a screening visit (baseline) will be performed at the day of admission to the study center. Since a retinal detachment is an emergency, which is unpredictable and which should be treated as soon as possible, a scheduled screening cannot be performed in advance. Thus, the screening is carried out following the emergency admission to the trial centers. This will include a full ophthalmic examination with slit-lamp/indirect biomicroscopy, and a structured interview, which will include questions on coexisting ocular pathology and previous ophthalmic surgical procedures to confirm that all inclusion and exclusion criteria are satisfied, will be conducted by a member of the study group. Furthermore, urine pregnancy tests will be performed in women of childbearing age and potential. Clinical findings documented as part of the routine clinical care at the time of screening may be used to populate data in the baseline case report forms (CRFs) and used as part of the study data (if collected within 5 days prior to PPV including IMP application for retinal detachment repair). This information may be collected prior to informed consent for enrolment into the trial as no additional intervention is performed outside routine clinical care. Patients will be informed in detail about the purpose of the study as well as the risks and benefits. Written informed consent will be obtained prior to study-specific measures. Postoperative study visits will not differ from the routine schedule for vitreo-retinal procedures at the study site for the first 3 months. The first postoperative visit is between days 1 and 5, the second postoperative visit 6 weeks postoperatively (± 10 days), and the third 12 weeks postoperatively (± 10 days).

**Fig. 2:**
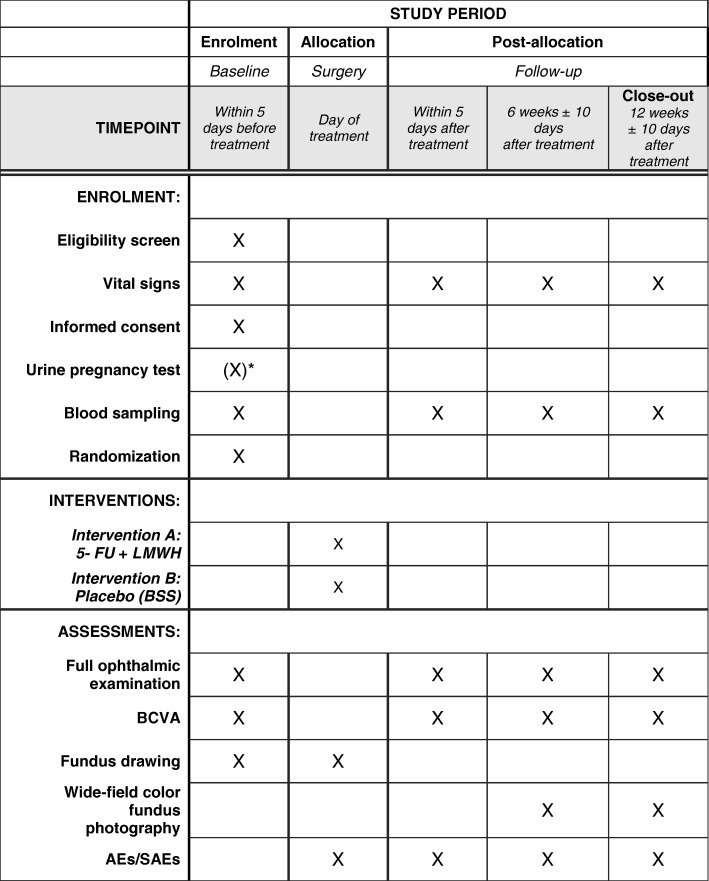
Schedule of enrolment, intervention, and assessments. Abbreviations: *5-FU* 5-fluorouracil, *AE* adverse event, *BCVA* best-corrected visual acuity, *LMWH* low-molecular-weight heparin, *SAE* serious adverse event. *Human chorionic gonadotropin (hCG) urine test: For all female patients of childbearing age

At each preoperative and postoperative study visit, again a full ophthalmic assessment will be completed and this will include slit lamp biomicroscopy (with indirect binocular ophthalmoscopy when required) and recording of parameters, including ETDRS visual acuity, Goldman applanation tonometry, anterior segment assessment, and retinal attachment status. Fundus drawing (at screening visit and surgery) will be performed to record preoperative and direct postoperative retinal status. Color fundus photography (nine-field or wide-angle) will be performed 6 and 12 weeks postoperatively for endpoint assessment (Fig. 2).

In case of any relevant complications in the study eye requiring re-surgery, additional unscheduled study visits over the trial period can become necessary. For unscheduled visits, CRFs identical in content to the regular study visit CRF will be completed and included in the data analysis upon completion of the study.

Following the final study visit at 12 weeks, participants will be discharged back to the care of their admitting consultant or will be under the care of a more appropriate specialist.

If a patient is lost to follow-up (i.e. at least not attending visit 5), then the patient’s resident ophthalmologist will be contacted after the 12-week follow-up period in order to obtain information about the events after the last regular visit unless prior permission to do so is refused by the patient.

As surgery for retinal detachment and follow-up visits require highly specialized centers such as the trial sites, the adherence of the patients will be warranted.

Excluded medication or treatments during the trial period include the following: intraocular corticosteroids or other intraocular drugs in study eye and medications known to be toxic to the lens, retina, or optic nerve, including chloroquine/hydroxychloroquine, deferoxamine, ethambutol, phenotiazines, or tamoxifen, and application of further investigational drugs. Furthermore, any therapy with an immunosuppressant or chemotherapy and intraocular surgery in study eye that is not related to previous vitrectomy due to retinal detachment or its complications is permitted during the trial.

### Outcomes

#### Outcome measures and efficacy assessment

We defined anatomical and functional outcome variables. The primary outcome measure, PVR grade CP 1 or higher, is in accordance with a trial which used 5-FU and LMWH in high-risk patients and in which a reduction of PVR grade CP 1 of 50% was observed [7]. PVR grade CP 1 constitutes the need for surgery as tractional retinal detachment is imminent. Surgical success is defined by retinal re-attachment.

BCVA serves as a secondary endpoint because it is an accepted endpoint for visual function.

All efficacy assessments are on the study eye and recorded in the electronic CRF (eCRF). Efficacy assessments will include primary and secondary endpoints (both anatomical and functional evaluations). For assessment of the primary endpoint defined as PVR grade CP 1 or higher (yes), fundus photos will be taken 6 and 12 weeks after treatment. Primary endpoint and secondary endpoints, including PVR grade CA 1 or higher, retinal re-attachment and grade of PVR, and number and extent of re-surgeries, will be assessed by fundus photos (6 and 12 weeks) and documentation of surgeons (regarding re-surgery during the trial period) evaluated by an external endpoint committee (EPC). BCVA of study eye will be recorded in the eCRF, and the occurrence of AEs related to the study drug will be assessed by AE/serious adverse event (AE/SAE) reporting.

#### Appropriateness of assessments

BCVA assessment using ETDRS-like visual acuity testing charts is a standard assessment. The same applies for the fundus photography. Both assessments are clinical standard.

The ETDRS score is generally recognized as reliable, accurate, and relevant in BCVA assessment. Fundus photographs will be graded by an EPC to ensure a uniform, standardized evaluation and assessment. Grading is performed in accordance with the updated Classification of the Retina Society [20].

#### Endpoint committee

An EPC is established in order to evaluate the incidence of the primary endpoint in accordance with the classification of retinal detachment with proliferative vitreoretinopathy [21]. Some secondary endpoints will also be evaluated by the EPC. The EPC is composed of four independent and fully blinded members. The EPC members are experienced ophthalmologists and vitreo-retinal experts. The evaluation of the endpoint is performed on the basis of fundus photos that are taken at two different time points during the study. In case of any necessary re-operation related to previous retinal detachment or performed vitrectomy (i.e. retinal re-detachment) within 12 weeks after treatment, records of operation documentation (eCRF) will be reviewed. Furthermore, pre- and intra-operative fundus drawings of revision surgeries will be reviewed. The EPC will assess whether PVR development was the most likely reason for the additional revision surgery, whether the retina was attached prior to revision surgery, and whether revision surgery was categorized as serious. At least two members of the EPC will assess each single photo and documentation of revision surgery by applying the endpoints in regular meetings. The individual members will meet by appropriate means and together assess the provided data.

### Participant timeline

Each individual will be followed up for 3 months postoperatively (last study visit 12 weeks ± 10 days postoperatively).

#### Proposed overall timescale

Trial start: October 2016.

Projected trial end: October 2020.

Trial duration: 48 months.

Duration of each patient’s participation: 3 months.

### Sample size

#### Determination of trial size

Hoerster et al. reported a risk of 40% (8/20, 95% confidence interval (CI) 21.9–61.3%) for PVR grade CP 1 in a high-risk population (flare ≥15 pc/ms) [14]. Asaria et al. reported a risk of 26.4% (23/87, 95% CI 18.3–36.6) for PVR in the control group [7]. Therefore, we assume an incidence of PVR grade CP 1 of 35% in the control group. In the trial conducted by Asaria et al., the reduction of PVR events achieved about 50% (relative risk (RR): 0.48, 95% CI 0.25–0.92) [7]. Given a reduction of 33% (1/3) as clinically relevant (i.e. an RR of 2/3, or 0.6667), the incidence of PVR in the experimental group of our trial would be 23.33%. With a Pearson’s chi-squared test in a group-sequential study design with one interim analysis after half of the recruited patients and boundaries of O’Brien/Fleming, a two-sided type I error of 5%, and a power of 80%, 478 patients in total (239 in each group) are needed in order to show a significant result. Fisher’s exact test and Pearson’s chi-squared test with Yates’s continuity correction are too conservative and therefore have not been applied [22, 23].

Simulating the effect of the stratification by surgeon (assuming 24 surgeons with about 20 patients), an additional 5% of patients is needed to achieve the same power. This is in accordance with the results presented by Donner [24]. Therefore, 478/(1–0.05) = 504 patients need to be recruited, excluding any potential dropouts. It is expected that most of the patients are compliant and only a small number of patients (5%) will drop out (lost to follow-up). When the sample size for the dropouts is adjusted by using the formula given by Donner, then 504/(1–0.05)^2^ = 560 patients (280 per treatment arm) will be necessary for recruitment [24]. Sample size calculations were performed by using R, version 3.1.3, package gsDesign.

### Recruitment

Patient recruitment was not started before approval of the competent ethics committee (institutional review board, or IRB). IRB approval was obtained (IRB no. 16–192). At each trial site, the clinical trial does not commence prior to approval of the competent local ethics committee concerning the suitability of the trial site and the qualifications of the investigators. All 560 patients will be identified and recruited from emergency referrals at the trial sites.

### Assignment of interventions

Patients are randomly assigned to either the verum arm or placebo arm by using a 24-7 internet online randomization tool: TEN-ALEA (https://prod.tenalea.net/zkskoeln/dm/). In a previous study, results showed that the surgeon had a considerable influence on the outcome of surgery [25]; therefore, the randomization is stratified by the surgeon. The randomization list was generated by using permuted blocks of varying sizes and by a statistician independent of the trial team. In the rare case of unavailability of the service, a fax-based fallback procedure is to be used.

#### Blinding (masking)

Participants and all study team members are completely masked to the treatment allocation in order to avoid any bias regarding surgical management and postoperative treatment. Owing to identical appearance, the IMP components cannot be distinguished.

Unblinding at the request of the investigator should occur only in the event of an emergency or AE for which it is necessary to know the study treatment in order to determine an appropriate course of therapy for the subject. Unblinding is then carried out by the investigator, who has to inform the CTCC about the event of unblinding immediately (within 24 h at the latest).

For every suspected unexpected serious adverse reaction (SUSAR) occurring during the specified reporting period, treatment unblinding of the individual trial patient will be performed by CTCC before reporting the event to the ethics committee, the competent authority, and the data monitoring committee (DMC).

#### Withdrawal

Participants can withdraw at any time during the trial at their own or their legal representative’s request without providing a reason and without any personal disadvantage. A participant may also be withdrawn if, on the basis of the investigator’s judgment, continuation of the trial may be detrimental to the participant’s health. Other possible reasons for the investigator to discontinue or modify a patient’s trial participation include relevant intra- or post-surgical complications (i.e. endophthalmitis) or other uncontrolled ophthalmologic disorders not related to previously performed vitrectomy or to trial drug application. Reasons for all withdrawals will be recorded in the patient’s medical files and their CRFs. The analysis will include a listing of all patients who prematurely terminate the trial, indicating the reason for discontinuation and reporting the data available on patient characteristics as well as on prespecified outcome parameters.

### Data collection methods and data management

All clinical documentation (source data) and data arising from the trial are to be kept by the investigator/trial site and be available for review by the clinical research associate.

The eCRFs were designed and produced by the sponsor and data manager of the CTCC. The final version was approved by the sponsor. It will be the responsibility of the investigators to ensure the accuracy of all data entered on the CRFs. A delegation log at each trial site is used to identify all trial personnel with responsibilities for data collection and handling, including those who have access to the trial database.

Plausibility checks are run during data entry, thereby detecting discrepancies immediately. CTCC Data Management has conducted further checks for completeness and plausibility and will clarify any questions with the trial sites electronically via the trial software. These electronic queries have to be answered by the trial site without unreasonable delay.

The database is integrated into a general IT infrastructure and safety concept with a firewall and backup system. The data are backed up daily. After completion and cleaning of data, the database is locked and the data exported for statistical analysis.

Central quality control located at the data management facility provides regular reports to provide information to project management to identify trial sites that might benefit from additional quality assurance measures such as risk-adapted monitoring. Reports provide, for example, trial site–based information regarding quality of eCRF documentation, query response time, or missing data.

### Data protection

The provisions of data protection legislation are observed. It is ensured by the sponsor that all investigational materials and data will be pseudonymized in accordance with data protection legislation before scientific processing.

Trial subjects are informed that their pseudonymized data will be passed on in accordance with provisions for documentation and notification pursuant to § 12 and § 13 of the Good Clinical Practice (GCP) Regulations to the recipients described there. Subjects who do not agree that the information may be passed on in this way will not be enrolled in the trial.

### Statistical methods

PRIVENT is a group-sequential trial with an interim analysis after half of the patients will have been recruited. The incidence of PVR grade CP 1 or higher after 12 weeks will be compared between the groups by using the conservative boundaries of O’Brien and Fleming. Therefore, the trial has to be stopped after the first stage if the *P* value of the Mantel–Haenszel test is not more than 0.0052. If the trial continues, the sample size of the second stage can be adopted. In case of continuation after the interim analysis, the *P* values of both stages are combined by using the inverse normal method by Lehmacher and Wassmer [26].

During the interim analysis, only the statistician, who provides the analysis, and the DMC members will have unblinded access to the data and results. The DMC members will provide advice regarding the continuation of the trial. If the study continues no further, information about the results (e.g. trends) will be given to the study personnel. They will only be informed to continue recruitment.

The primary analysis set is the modified intention-to-treat (mITT) population. It includes all enrolled and randomly assigned patients who received the initial surgery. Patients will be analyzed in the assigned treatment groups regardless of the actual received treatment. Patients lost to follow-up within 12 weeks are counted as treatment failures in the analysis if no information about PVR status can be obtained.

The secondary analysis set is the per-protocol (PP) population. It is a subset of the mITT population and includes all patients who received the trial interventions as assigned, who were treated and observed according to protocol, and who had no major protocol violations. Prior to the interim and final analysis, a blind review of all protocol violations will be carried out by the lead investigator (or representative).

The primary endpoint of the trial is the occurrence of PVR grade CP 1 within 12 weeks. The null hypothesis H_0_ is “the PVR grade CP 1 incidence is equal in both treatment groups (verum, placebo)”.  It will be tested by application of the Mantel–Haenszel test [27] accounting for the stratification by surgeon. The primary analysis is performed by using the mITT population. Missing values are assumed to be missing at random. For the primary analysis, a missing primary endpoint is considered a treatment failure. RRs and absolute risk reductions (with CIs and *P* values) will be given for the overall effect between the trial groups and within the strata.

Secondary endpoints will be evaluated by descriptive methods. Numerical data will be summarized by number of patients, mean, standard deviation, median, first quartile, third quartile, and minimum and maximum; categorical data will be summarized by number and percentage of patients. If *P* values are computed for the secondary parameters, no adjustment for multiplicity and interim analyses will be taken into account. Therefore, no confirmatory test decisions are possible with these *P* values. *P* values of not more than 0.05 (5%) are considered statistically significant.

For all primary and secondary parameters, descriptive statistics will be given overall and for each treatment group. Moreover, results of both stages and overall will be given.

The safety population includes all randomly assigned patients who received the initial surgery. Analysis is according to the treatment received. The analysis includes calculation and comparison of the rates of specified complications (see secondary endpoints) and SAEs as well as of severity and relationship to intervention and graphical display of the time course.

Furthermore, statistical methods are used to assess the quality of data and the homogeneity of intervention groups. Analyses will be carried out by using SAS version 9.3 (SAS Institute Inc., Cary, NC, USA) or IBM SPSS Statistics 23 or higher (IBM, Armonk, NY, USA).

### Monitoring

Monitoring is performed risk-adapted. Risk groups are classified according to data quality, compliance, or problems with the study implementation. Depending on the risk group, the study sites are visited at different frequencies. Each site received a site selection visit to ensure the presence of sufficient capacity and equipment. Regular visits are performed throughout the trial, beginning with an initiation visit prior to study start, and a close-out visit will be performed at each trial site.

### Adverse events and safety reporting

Safety reporting will adhere to the sponsor’s standard operating procedures. There is an external DMC, which has an agreed charter. It consists of two physicians and a statistician who are not involved in the conduct of the trial. The task of the DMC is to oversee the safety of the trial subjects in the clinical trial by periodically assessing the safety of the trial therapy. They will meet every six months or on an ad-hoc basis as required.

Expected AEs are development of PVR, retinal re-detachment, cataract, raised IOP, and further surgery. Unexpected AEs will include endophthalmitis, systemic illness, and ocular vascular occlusion.

Furthermore, an AE of special interest (AESI) has been defined in order to evaluate visual impairments due to potential toxic effects of the IMP at an early stage. The AESI will be handled as an SAE and has been defined as the lack of recovery of visual acuity (BCVA) to 0.3 log MAR/0.5 decimal or better and/or decrease of visual acuity (BCVA) to > 0.3 log MAR/< 0.5 decimal (compared with the last assessment of visual acuity prior to the most recent assessment) lasting more than 1 h during the postoperative course without morphological correlate in study eye that presented with primary rhegmatogenous retinal detachment with macula-on status prior to surgery.

All AEs are documented in the trial patient’s medical records and the eCRF, including date and time of onset and resolution, severity, causal relationship with IMP/study treatment, seriousness, and interruption or withdrawal of study treatment and other measures taken.

Regardless of whether a causal relationship between the AE and the IMP is suspected, trial patients who develop AEs must be monitored until all symptoms have subsided, pathological laboratory values have returned to pre-event levels, a plausible explanation is found for the AE, the trial patient has died, or the study has been terminated for the trial patient concerned. This information must be verified on the basis of the source data.

Regardless of the assumed causal relationship, every SAE must be documented in the eCRF and on an SAE form, which has to be sent to the sponsor immediately (no later than 24 h after being aware of the SAE). All SAEs are assessed by the sponsor and PI with regard to seriousness and causality. The expectedness assessment is carried out by the sponsor. If an AE is “serious”, “related”, and “unexpected”, the criteria for an expedited report (SUSAR) are fulfilled. Every SUSAR that becomes known in this clinical trial is reported to the competent authorities and the responsible ethics committee, to the PI of each participating trial site, the DMC chairman and to the PI of all clinical trials investigating with the same active substance (IMP) by the sponsor.

### Ethics and dissemination

Before the start of the clinical trial, all necessary documentation has been submitted to the competent supreme federal authority for approval (Federal Institute for Drugs and Medical Products, Bundesinstitut für Arzneimittel und Medizinprodukte [BfArM]) and the local IRBs of all attending centers (Appendix includes a list of all approved attending centers and local IRBs). Favorable opinions have been received (overall IRB no. 16–192). The state authorities in each federal state in which the trial will be conducted were also notified. Further competent supreme federal authority, local authorities, and ethics committees will be informed about the end of study.

The study is conducted in accordance with the International Conference on Harmonization for Good Clinical Practice (ICH-GCP). The study will comply at all times with the Declaration of Helsinki (2000). The results of this study will be submitted for publication in peer-reviewed medical journals regardless of whether the findings are in favor of the trial intervention.

## Discussion

The incidence of primary RRD varies, according to different studies, between 10 and 13 per 100,000 [28]. PVR is characterized by the formation of fibrovascular scars leading to secondary tractional retinal detachments, which often require multiple extensive surgical interventions to finally achieve retinal re-attachment [1, 29, 30]. Observational studies of patients with PVR showed that over 80% of patients become either severely visually impaired or blind, according to the definition of the World Health Organization, in the course of the disease [3]. There are no prophylactic agents against PVR routinely in use. 5-FU and LMWH would be the first treatment reducing the risk for PVR and thus the risk for irreversible blindness of patients. Pre-selection of patients by laser flare photometry could confine the use and possible side effects of treatment to high-risk patients for PVR.

The intervention comprises a standard-of-care PPV with either intraoperative adjuvant application of a combination of 5-FU and LMWH or placebo (BSS) via intraocular infusion during routine PPV in patients with primary RRD. The concentrations of 5-FU and LMWH are 200 μg/mL and 5 IU/mL, respectively, as have been investigated in previous clinical trials by Asaria et al., Charteris et al., and Wickham et al. (Table 2) [7, 9, 11, 17]. The verum/placebo containing infusion will be used for a maximum of 60 min. If surgery takes longer, infusion will be changed to normal BSS. Retinopexy by endolaser will be used for retinal breaks; cryopexy is allowed for retinal breaks inaccessible to endolaser. The intraocular endotamponade can be expanding gas or silicone oil. All of these conditions are in accordance with the two previously described trials using 5-FU and LMWH [7, 9, 17]. Additional scleral buckling with an encircling band (360°) or plombe is permitted.

**Table 2:** Specification of endpoints

Endpoint	Measurement variable	Analysis metric	Method of aggregation	Time (after initial surgery)
Primary endpoint:				
PVR grade CP 1 (or higher)	Full thickness retinal fold ≥1 clock hour	- Value (yes/no)	- Proportions	- Within 12 weeks
Secondary endpoints:		-	-	-
PVR grade CP 1 (or higher)	Full thickness retinal fold ≥1 clock hour	- Value (yes/no)	- Proportions	- Within 6 weeks
PVR grade CA 1 (or higher)	Full thickness retinal fold ≥1 clock hour	- Value (yes/no)	- Proportions	- Within 6 weeks and 12 weeks
Degree of PVR	PVR grade (CA 1–12, and/or CP 1–12)	- Value (yes/no) - Count (clock hours)	- Proportions - Means	- Within 6 weeks and 12 weeks
Best corrected visual acuity	ETDRS letters	- Count	- Means	- Within 6 weeks and 12 weeks
Retinal re-attachment after primary intervention	Retinal attachment in all 4 quadrants	- Value (yes/no)	- Proportions	- Within 6 weeks and 12 weeks
Number of retinal re-detachments	Number of retinal re-detachments if present, re-detachments due to PVR	- Count (>0) - Value (yes/no)	- Proportions	- Within 6 weeks and 12 weeks
Number and extent of surgical procedures to achieve retinal attachment	Number of procedures number of intraoperative procedures number of serious/ complex procedures	- Count (>0) - Value (yes/no)	- Proportions	- Within 12 weeks
Occurrence of at least one drug-related adverse event that affects the study eye	Drug-related adverse event that affects the study eye	- Count	- Proportions	- Within 12 weeks

Abbreviations: *CA* grade C anterior, *CP* grade C posterior, *ETDRS* Early Treatment Diabetic Retinopathy Study, *PVR* proliferative vitreoretinopathy

BSS equally packaged as the verum 5-FU and LMWH will serve as placebo to provide blinding of the trial. Usage of placebo is justifiable since no standard treatment for the prevention of PVR currently exists.

Primary analysis is according to mITT. Patients lost to follow-up within 12 weeks are counted as “events” if no information about PVR status can be obtained. This shall guard against overly optimistic results and minimize possible attrition bias. Throughout the trial period, all patients fulfilling main inclusion criteria are screened and documented. Monitoring of the trial is carried out in accordance with international GCP guidelines. Patients with other ophthalmologic disorders leading to blood-brain barrier breakdown and to additional risk for PVR are excluded to homogenize the sample.

In summary, according to the recommendation of the COCHRANE collaboration, the present trial uses intraoperative intravitreal 5-FU and LMWH as a prophylactic therapy in high-risk patients with primary RRD aiming to reduce the incidence of PVR in the group that receives the trial drug. Using laser flare photometry to determine high-risk patients for PVR, this trial will test the effectiveness of a simple treatment to prevent PVR (Additional file 1).

### Trial status

The authors confirm that the trial was registered prior to the start of randomization (EudraCT no.: 2015-004731-12, registered October 21, 2015; ClinicalTrials.gov Identifier: NCT02834559, registered July 12, 2016) and in active recruitment at the time of manuscript submission.

### Additional file

Additional file 1: SPIRIT 2013 Checklist. (DOC 101 kb)

## Appendix

1. List of trial sites and corresponding institutional review boards

## Abbreviations

AE: Adverse event
AESI: Adverse event of special interest
BCVA: Best corrected visual acuity
BSS: Balanced salt solution
CI: Confidence interval
CRF: Case report form
CTCC: Clinical Trials Center Cologne
DMC: Data monitoring committee
eCRF: Electronic case report form
EPC: Endpoint committee
ETDRS: Early treatment diabetic retinopathy study
IMP: Investigational medicinal product
IOP: Intraocular pressure
IRB: Institutional review board
LMWH: Low-molecular-weight heparin
MAR: Magnification requirement
mITT: Modified intention-to-treat
pc/ms: Photon counts per millisecond
PI: Principal investigator
PP: Per-protocol
PPV: Pars plana vitrectomy
PRIVENT: Prevention of Recurrent Urinary Tract Infection in Children with Vesicoureteric Reflux and Normal Renal Tracts
PVR: Proliferative vitreoretinopathy
ROC: Receiver operating characteristic
RR: Relative risk
RRD: Rhegmatogenous retinal detachment
SAE: Serious adverse event
SUSAR: Suspected unexpected serious adverse reaction
